# How We Are Poisoned

**Published:** 1881-06

**Authors:** 


### HOW WE ARE POISONED.

At a recent meeting of the Lancaster, Pa., Agricultural Society, Dr. C. A. Greene read a paper on the above subject in which we find the following:

Thousands of persons die every year from poisons taken into the stomach. I propose briefly to show in what manner it is done, and also to show that thousands of persons also suffer pains, some of them almost indescribable, from the absorption of poisons into the body. On the outside of the body are millions of little holes called absorbents, which have the power like a suction-pump of drawing into the body almost anything that may come in contact with the skin. Hence it is a self-evident fact that under no consideration should poisons of any kind be handled nor should they be taken into the alimentary canal. The object of a man or animal's stomach and intestines is to convert food into blood, and any foreign substance in these organs acts (like a splinter in the flesh) irritantly. Hence they are contra-indicated. Newspapers throughout our commonwealth often publish receipts and items on physiology that are truthless and worthless, and often exceedingly injurious. In a March number of the Philadelphia *Record* sulphate of zinc and foxglove (or digitalis) are called a sure remedy for smallpox, and yet both of them are powerful poisons; one grain of foxglove, which is the 1-480th part of an ounce, has been known to produce vertigo, extreme pains, dimness of vision, and a reduction of the pulse from 80 to 40 beats a minute. In the same issue was the following receipt:

"A solution of oxalic acid is the best for scouring and polishing copper. Finish with whiting."

Now as editors are not chemists or physicians, why will they in this reckless manner give such statements to their readers? The blacksmith who never saw an astronomical instrument does not force his crude conceptions of celestial bodies upon the people. Oxalic acid is also a very dangerous poison, and only a few grains of it taken into the stomach will produce disastrous symptoms and death, and merely handling it may introduce into the system sufficient to produce thousands of unnecessary pains and aches. It should never be found in your home; it is as dangerous as a rattlesnake.

### COPPER UTENSILS.

Many farmers do a large amount of cooking for themselves and their cattle, poultry, etc., in copper and brass kettles. Any of them when not used for a time are lined with verdigris, called in the books subacetate of copper, also oxide of copper, and it is soluble in water and is a virulent poison. Brass kettles are made from copper and zinc. Any acid will always act upon metals. If you stew apples, cranberries, tomatoes, or any fruit or vegetable that is of an acid nature, the acid eats or corrodes the copper or zinc and forms usually acetate of copper or zinc. No matter how small the quantity swallowed it is a foreign substance, as well as poisonous and produces indigestion. The acid of apples is called malic or sorbic acid, and if it comes in contact with copper, zinc, lead, or tin, will produce malatate of copper, zinc, lead and tin. The fermentation of apples or cider, made from apples, produces vinegar, which is dilute acetic acid, and it will also produce the same chemical changes if it has the opportunity, and the results will be acetate of copper, acetate of zinc, lead or tin. When the milk becomes sour it produces lactic acid, which will act in the same manner as the two acids, and form lactate of copper, lead, zinc and tin, and all of these metals are poisonous, and every one injures the health of the individual who has eaten them in his or her food. Dyspepsia in some of its forms, paralysis, neuralgia, and affection of the organs of the body, are the sequences. I would as soon have a copperhead snake in my house as a brass or copper utensil for cooking purposes. If they are scoured ever so clean, the acid will act upon them even more readily. It is a common occurrence when pickles become a little changed in the spring, to put pickle and vinegar in a copper or brass kettle and boil them for a time and they come out much improved in appearance and handsomely greened. This bright color is acetate of copper. Tin vessels also lose their luster by long exposure, and form a poison called oxide of tin. Lead pipes have been used for many years to convey drinking water ; if it stands for any length of time in the pipe the oxide of lead is formed, and any one drinking it is poisoned.

The quail and partridge in the cold winter months eat poison berries, and in this way they contaminate their flesh and injure the health of the one who eats it. Acetic acid is distilled vinegar. If you take one pint of acetic acid and seven pints of water and unite together, you have eight pints of vinegar.

SOAP.

Some soap makers, regardless of the consequences, take the tallow or fat of diseased animals and make them into soap. The unchanged virus is absorbed into the body while being used for washing purposes. If you cook lemons in a brass or copper kettle, the acid of the fruit, called citric, will act upon the metals in the same manner and form citrate of copper, zinc, etc.

HAIR BRUSHES.

Many persons use the hair brush of another individual, or the barber uses upon a hundred or a thousand heads the same brush. If any of his patrons have tetter, eczema, syphilis, or other skin disease, it can be readily conveyed to any one whose head is briskly rubbed with it. In the above and many other ways are poisons conveyed into the body, and the victim of the virus may suffer all his life from the effects. I have brought for inspection some of these poisons, and to show how small a quantity of copper will by the laws of affinity make itself known, I propose to add one drop of a solution of nitrate of copper to one hundred drops of water, and then add one drop of aqua ammonia to the colorless liquid, and it will at once become beautifully blue. I will conclude by saying that there is a friend of mine in this city who has over 100 tumors on his body occasioned by his handling paints.

At the close of his essay Dr. Greene made a number of chemical experiments with the poisons referred to in the essay.

Mr. Engle said it was news to him that the souring of milk in tin cans produced a poisonous acid, and yet there seemed to be no doubt it would do so.

In answer to questions, Dr. Greene said that tin was a less dangerous metal to be brought in contact with food than zinc, brass or copper. Iron vessels may be safely used as cooking utensils, as when iron taken in proper proportions is not injurious; but people usually get enough of it in the food cooked in iron vessels, without taking it as a medicine.

Mr. Linville believed there was great danger of being poisoned by the use of milk if kept in tin pans, and thought dairymen made a great mistake in substituting tin cans for the old-fashioned earthen crocks. If vessels of pure block tin were used, and were kept scrupulously clean, there might not be much danger in using them, but unfortunately, the so-called tinware contained a large proportion of lead which is more readily decomposed by acids than tin and is also a much more virulent poison. He also spoke of the danger of poison from boiling apple butter in copper-kettles; and yet he and everybody else use copper-kettles for this purpose. As people will have apple butter, he advised that the kettle be scoured scrupulously clean, that the cider be immediately put into and heated as soon as possible, and that all the apple butter be removed from the kettle before it cools, as the decomposition of the copper and the formation of the poison goes on much more rapidly when the acid of the apple butter is cold than when it is hot.—*Scientific American Supplement.*

